# ﻿Taxonomic and nomenclatural history of *Neckera* (Bryophyta, Neckeraceae), including reinstatement of *Rhystophyllum*, the correct name for a segregate of this genus

**DOI:** 10.3897/phytokeys.267.171699

**Published:** 2025-12-02

**Authors:** Ryszard Ochyra, Vítězslav Plášek, John C. Brinda

**Affiliations:** 1 National Biodiversity Collection, W. Szafer Institute of Botany, Polish Academy of Sciences, Lubicz 46, 31-512 Kraków, Poland Polish Academy of Sciences Kraków Poland; 2 Department of Biology and Ecology, University of Ostrava, Chittussiho 10, 710 00 Ostrava, Czech Republic University of Ostrava Ostrava Czech Republic; 3 Institute of Biology, University of Opole, Oleska 48, PL‐45‐052 Opole, Poland University of Opole Opole Poland; 4 Missouri Botanical Garden, 4344 Shaw Boulevard, Saint Louis, MO 63110, USA Missouri Botanical Garden Saint Louis United States of America

**Keywords:** *
Alleniella
*, *
Exsertotheca
*, generic names, infrageneric taxa, Musci, nomenclature, *nomina generica conservanda*, pleurocarpous mosses, Madrid Code, taxonomy, unitary designations of species

## Abstract

The complex taxonomic history of the moss genus *Neckera*, whose name was conserved at the Paris Congress in 1954, is reviewed. The issue of typifications of moss generic names by W. Ph. Schimper in 1860 is examined in detail and it is concluded that these typifications comply with the provisions of the "*International Code of Nomenclature for algae, fungi and plants*". Schimper was the first to typify *Neckera* with *N. pennata*, so it is unnecessary to treat this as a conserved type. The unitary designations of moss species proposed by Jakob Friedrich Ehrhart in his exsiccata "*Phytophylacium Ehrhartianum*", published between 1780 and 1785, are reviewed. It is concluded that two of these designations, *Diphyscium* and *Paludella*, are currently in use, having been validly published in the early nineteenth century. The same applies to *Rhystophyllum*, another of Ehrhart’s unitary designations, which was validated as a subsection of *Neckera* by C. Müller in 1850 and, subsequently, first elevated to the rank of section by Mitten in 1869, rather than Braithwaite in 1905 as listed in "*Index muscorum*". Finally, E. Britton raised this infrageneric taxon to the rank of genus in 1904. *Rhystophyllum* is here reinstated from obscurity, since it is homotypic with the generic name *Exsertotheca* that was recently introduced for a segregate of *Neckera*. Accordingly, three new combinations are proposed: *Rhystophyllum crispum*, *Rh. intermedium* and *Rh. baeticum*. Neckera subsect. Leiophyllum is regarded as a heterotypic synonym of *Alleniella*, another recent segregate of the formerly broadly circumscribed genus *Neckera*.

## ﻿Introduction

The rapid advancement of phylogenetic taxonomy in recent decades has led to the increasing subdivision of large, traditionally recognised genera. These genera were originally circumscribed using methods of classical descriptive taxonomy, based primarily on morphological and anatomical features. As a result, many have since been split into multiple, smaller genera. The newly-delineated genera are typically assigned new names, a practice permitted under the "*International Code of Nomenclature*" (ICN; Turland et al. (2025)). However, it occasionally emerges that some generic names, validly published in the distant past, but subsequently forgotten, still meet all the necessary criteria for use and may be appropriately applied to these newly-recognised segregates.

This article examines one such overlooked genus which, despite having fallen into obscurity, warrants reinstatement and application to one of the segregates of *Neckera* Hedw., a large and well-known genus of pleurocarpous mosses in the family Neckeraceae Schimp. *Neckera* has recently been subdivided into several smaller genera (Enroth 1991; Olsson et al. 2009a, 2009b, 2011, 2012, 2016; Enroth et al. 2022), for which new names have been proposed. One of these segregates, *Exsertotheca* S.Olsson, Enroth & D.Quandt, includes a common European calcicolous species previously known as *N. crispa* Hedw. This article reviews the taxonomic and nomenclatural history of the genus *Neckera*, with particular emphasis on its infrageneric classification. In many cases, the names of infrageneric taxa can be adopted for segregate genera through a simple change in status, thereby eliminating the need to coin entirely new names and to provide separate diagnoses and descriptions.

## ﻿Brief taxonomic history of *Neckera*

### ﻿From Hedwig to Müller Hallensis

**Figure 1.**
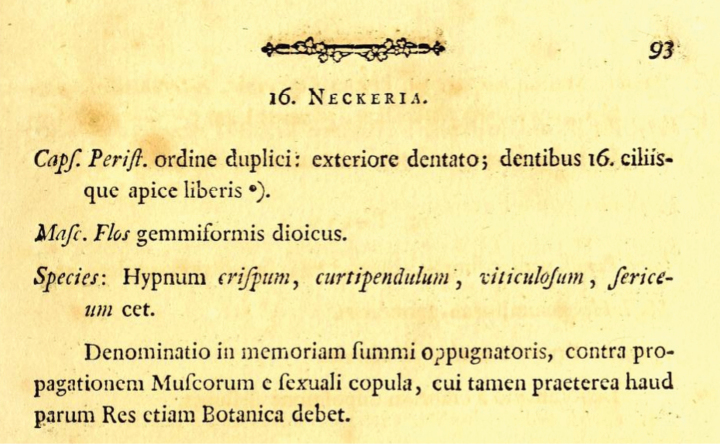
The original publication of the generic name *Neckeria* Hedw. (from Hedwig 1782).

*Neckera* Hedw. is amongst the earliest generic names in muscology, first introduced by Hedwig (1782) under the orthographic variant *Neckeria* Hedw. (Fig. 1). The name commemorates Noël Martin Joseph de Necker (1730–1793), a French-born botanist and bryologist who served as the personal physician to the court of the Electoral Palatinate in Mannheim, Germany (Frahm and Eggers 2001). Although Necker strongly opposed Hedwig’s view that mosses reproduce sexually, asserting instead that they do not (Schofield 1985), Hedwig nonetheless recognised Necker’s substantial contributions to botany, a fact he noted in justifying his choice of this eponym.

Originally, four species were placed in *Neckeria*, including *Hypnum crispum* L. A decade later, Hedwig (1792) corrected the orthography of this name to *Neckera* and expanded the genus to include nine additional species, such as *N. pennata* Hedw. and *N. pumila* Hedw. However, neither *Neckeria* Hedw. (1782) nor *Neckera* Hedw. (1792) constitute validly published names under modern nomenclatural rules, as both appeared prior to 1801, the year Hedwig (1801) published "*Species muscorum frondosorum*". This work is the designated starting-point for moss nomenclature (excluding Sphagnaceae) under the ICN (Turland 2013; Turland et al. 2025), in contrast to most other groups whose nomenclature begins with Linnaeus (1753).

**Figure 2.**
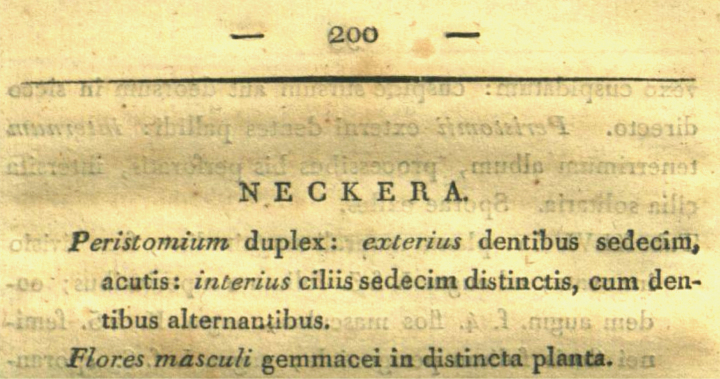
The original publication of the generic name *Neckera* Hedw. (from Hedwig 1801).

Hedwig (1801) validated the generic name *Neckera* by giving a short diagnosis which included very general features of the peristome, namely that it is double and consists of 16 teeth of the outer peristome alternating with 16 distinct ciliate processes of the inner peristome (Fig. 2). The genus was quickly and widely accepted; however, under this broad circumscription, *Neckera* could encompass most pleurocarpous mosses that were primarily classified in the former order Isobryales (= Leucodontales). Indeed, this was the case, as throughout the past two centuries, over 600 specific and about 100 varietal epithets, as well as over 100 binary designations, were combined with *Neckera* (Wijk et al. 1964).

One of the most striking examples of *Neckera* serving as a catch-all genus is found in the broad concept proposed by Müller (1850, 1851), published in September 1850 and supplemented in October 1851 (Stafleu and Cowan 1981). Müller assigned 199 species, known in the first half of the nineteenth century, to this single genus, subdividing them into nine sections and thirteen subsections, which highlighted its remarkable morphological heterogeneity. In modern classification systems, these species are now distributed across dozens of genera and multiple families of pleurocarpous mosses. In one case, the group even includes an acrocarpous species: *Dicnemoloma pallidum* (Hook.) Wijk & Margad. Of the 199 species included in Müller's (1850, 1851) synopsis of the genus, only 20 correspond to *Neckera* as currently circumscribed.

### ﻿Schimper’s concept of *Neckera* and its development

By a remarkable coincidence, just three months after publication of Müller’s (1850) concept of *Neckera* − in December 1850 − Schimper (1850a), in another monograph forming part of the monumental opus "*Bryologia europaea*" (Stafleu and Cowan 1976), presented a concept of this genus that was diametrically opposed to Müller’s. He assigned seven European species to the genus, including *N. pennata*, *N. pumila*, *N. crispa*, *N. oligocarpa* Bruch, *N. complanata* (Hedw.) Huebener, *N. sendtneriana* Schimp. and *N. philippeana* Schimp., all of which were described and illustrated in detail. In addition, he referenced five extra-European species: *N. menziesii* Hook. then known only from North America; *N. hornschuchiana* Müll.Hal. and *N. angustifolia* Müll.Hal. from Mexico; *N. chilensis* Schimp. from Chile; and *N. jamesonii* Taylor from Ecuador, but without comment. He also listed nine further designations in the protologue, including four from Africa, two each from Mexico and South America and one from Tasmania, all ascribed to W. Ph. Schimper. The species cited in the protologue roughly correspond to the two subsections distinguished by Müller (1850) within section ‘Euneckera ’ Müll.Hal., i.e. the type section of Neckera, namely subsect. Leiophyllum Müll.Hal. and subsect. Rhystophyllum Müll.Hal. This section encompassed a number of species that are now classified under other genera, mostly related to *Neckera* or the Neckeraceae, such as *Homaliodendron* M.Fleisch., *Porothamnium* M.Fleisch., *Neckeropsis* Reichardt, *Himantocladium* (Mitt.) M.Fleisch., *Calyptothecium* Mitt. and *Trachyloma* Brid.

Schimper’s (1850a) newly-proposed taxonomic concept of *Neckera* was quickly accepted in both Europe and North America, although the genus is not particularly species-rich in these regions. As exploration of tropical and subtropical areas advanced, new species of neckeroid mosses were discovered. These species often bore only superficial morphological similarities to *Neckera*, but due to the underdeveloped generic classification within the Neckeraceae, they were provisionally assigned to *Neckera* anyway (e.g. Mitten (1859, 1869); Dozy and Molkenboer (1863); Bescherelle (1872)). Consequently, many of these species were later transferred to other genera. In "*Adumbratio florae muscorum totius orbis terrarum*", published in the 1870s, Jaeger and Sauerbeck (1877, 1879) included 92 species and nine designations within *Neckera*. However, only 50 of those species could be retained in *Neckera* under the current taxonomic circumscription of this genus. Over the next quarter-century, the number of species assigned to *Neckera* grew to approximately 150. However, the number of species truly belonging to the genus in the strict sense increased to little more than a dozen (Paris 1896, 1900, 1905).

Finally, Brotherus (1906) published the first global taxonomic overview of the genus *Neckera* in Engler’s "*Die natürlichen Pflanzenfamilien*" series, providing general keys to the species. He recognised 127 species within the genus, which were organised into six sections. This concept differed significantly from Schimper’s (1850a) circumscription of *Neckera*, as nearly half of the species were later found to belong to what are now widely accepted as separate genera − *Neckeropsis* and *Himantocladium*. These species were formally transferred to the respective genera in the second edition of the moss treatment in "*Die natürlichen Pflanzenfamilien*" (Brotherus 1925), reducing the number of *Neckera* species to 89. This taxonomic framework remained influential throughout the twentieth century, although the number of recognised species continued to fluctuate. Names of many previously described species were synonymised with earlier ones, while others continued to be newly described. According to Crosby et al. (2000), *Neckera* comprised 71 species, with 27 of these remaining uninvestigated since their original description.

### ﻿Genus *Neckera* in the molecular era

A new chapter in the taxonomic study of *Neckera* began over the past two decades, marked by the integration of phylogenetic taxonomic methods, based on molecular analyses (Olsson et al. 2009a, 2009b, 2010, 2011, 2012, 2016; Enroth et al. 2022). This advancement led to a re-definition of the genus, which was subsequently divided into three segregates. Amongst them are *Alleniella* S.Olsson, Enroth & D.Quandt, comprising 15 species and *Exsertotheca* S.Olsson, Enroth & D.Quandt, containing three species (Olsson et al. 2011; Enroth et al. 2022). In addition, six monotypic genera have been newly established or reinstated to accommodate certain *Neckera* species. These include *Caduciella* Enroth (Enroth 1991), *Pengchengwua* S.Olsson, Enroth, Huttunen & D.Quandt (Olsson et al. 2016), *Enrothia* Ignatov & Fedosov (Fedosov and Ignatov 2019), as well as *Indoneckera* Enroth, *Taiwanobryopsis* Enroth and *Metaneckera* Steere (Enroth et al. 2022). Furthermore, eight species have been transferred to the genus *Taiwanobryum* Nog. (Olsson et al. 2010; Ma et al. 2018; Enroth and Shevock 2021) and ten species have been re-assigned to *Forsstroemia* Lindb. (Olsson et al. 2011; Enroth et al. 2019, 2022).

As a result of these taxonomic revisions, the genus *Neckera* currently comprises 39 extant and two fossil species (Brinda and Atwood 2025). Of the extant species, only 23 are widely recognised and accepted in taxonomic monographs, revisions and descriptive floras. The remaining 16 species are known solely from their original descriptions and have yet to undergo comprehensive taxonomic evaluation.

## ﻿Nomenclatural history of the generic name *Neckera*

### ﻿Conservation and former typifications

The generic name *Neckera* has a rather complex nomenclatural history. When Hedwig (1782) first published the name *Neckeria* for a newly-established genus of mosses, it was predated by an identical name used by Scopoli (1777) for a flowering plant in the family Papaveraceae. Even when later the spelling of this name was corrected to *Neckera* (Hedwig 1792, 1801), it was, from a nomenclatural perspective, merely an orthographic variant and, thus, considered a later homonym of *Neckeria* Scop. As such, it could not be validly used as a name for an accepted moss genus. To resolve this issue, *Neckera* Hedw. was formally proposed for conservation prior to the Sixth International Botanical Congress held in Amsterdam in 1935 (Dixon 1934). While this proposal was recommended by the nomenclature committee at that Congress (Dixon 1935; Magill 1993) and *Neckera* was placed in the list of "*Nomina generica conservanda*" in Appendix V of the "*Stockholm Code*" (Lanjouw et al. 1952), it was not formally acted upon until much later because no bryophyte names were formally conserved prior to ratification at the Paris Congress in 1954 (see below).

The typification of a generic name is fundamental to the taxonomic interpretation of a genus. This is particularly critical for older names, which often encompassed a wide array of diverse and, sometimes, unrelated species. One such name is *Neckera*, whose typification also has had a complex and convoluted history. According to Crosby (1968), Grout (1934) was the first to designate *N. crispa* as the type. However, Grout (1939) later “amended” this designation in favour of *N. pennata*. As noted by Brinda and Davidse (2020), this change appears to have been influenced by discussions amongst members of the nomenclature committee, who selected *N. pennata* as the type instead (Dixon 1939). However, this decision conflicts with Art. 10.5 of the "*Code*" (Turland et al. 2025), which would give precedence to the earlier designation of *N. crispa*.

Nevertheless, the revised typification was accepted by the General Committee and Advisory Board at the Eighth International Botanical Congress in Paris in 1954. Since the publication of the "*Paris Code*" (Anonymous 1954; Lanjouw et al. 1956), *N. pennata* has been listed as the type species of *Neckera*. Considering Grout’s earlier designation of *N. crispa* in 1934, *N. pennata* has been included in Appendix III of the "*Code*" as a conserved type since the "*Montreal Code*" (Lanjouw et al. 1961). Crosby (1968) opposed this outcome and proposed reinstating *N. crispa* as the generitype of *Neckera*, but his proposal was ultimately rejected by the Committee for Bryophytes (Florschütz 1973). Consequently, the recognition of *N. pennata* as a conserved type has achieved broad consensus (Wijk et al. 1964; Farr et al. 1979a; Greuter et al. 1993).

### ﻿Schimper’s typifications of moss generic names

In accordance with the delimitation of *Neckera* in "*Bryologia europaea*" (Schimper 1850a) and its subsequent usage, *N. pennata* Hedw. was formally designated as the type of this generic name by Schimper (1860) in the first edition of his "*Synopsis muscorum europaeorum*". This typification has largely been overlooked by bryologists, despite the relevant information appearing verbatim on the title page of the work: “*Accedunt tabulae VIII****typos genericos****exhibentes, ...*” [= “There are also eight plates showing **generitypes**” (*emphasis ours*)]. This critical phrase was first identified and interpreted in the mid-1990s by W. D. Margadant, who prepared a manuscript on Schimper’s typifications, which was submitted to Lindbergia. Pekka Isoviita of Helsinki was invited to serve as a referee for the manuscript and undertook a meticulous review, introducing numerous corrections and amendments. As a result, Margadant proposed that Isoviita be included as a co-author of the paper (Isoviita, personal communication to R. Ochyra *in litteris*, 23 February 1995; Margadant and Geissler (1995)). Unfortunately, the planned publication was never completed and published. However, typifications for four generic names, *Dicranella* (Müll.Hal.) Schimp., *Hylocomium* Schimp., *Pylaisia* Schimp. and *Homalia* Brid., were later published by Margadant and Geissler (1995) as part of formal proposals to conserve these names.

For three of these, conservation of the types was necessary to maintain current usage, which had been threatened by Schimper’s (1860) overlooked type designations involving different species. In the case of *Homalia*, however, the typification of the name was merely confirmed by Schimper (1860), since in "*Bryologia europaea*", Schimper (1850b) had used the term *Grundform*, in the German text only, as an equivalent of “type”. All these proposals were accepted by the Committee for Bryophyta (Zijlstra 1998, 1999) and the conserved generic names were subsequently included in Appendix III of the "*Saint Louis Code*" (Greuter et al. 2000), thereby formally endorsing Schimper’s (1860) typifications.

In addition to the article by Margadant and Geissler (1995), published in the November issue of Taxon, Volume 44, Margadant’s discovery was also acknowledged in the monograph on the genus *Racomitrium* Brid. in Poland (Bednarek-Ochyra 1995). Notably, this monograph was published even earlier, on 1 October 1995, than the aforementioned article. As mentioned above, the information regarding the typification of generic names in Schimper’s (1860) work had been communicated to R. Ochyra by P. Isoviita, who also granted permission for its inclusion in the monograph of *Racomitrium*. This case is of particular interest because Schimper (1860) designated *R. lanuginosum* (Hedw.) Brid. as the type, thereby rendering superfluous a later typification by Pfeiffer (1874), who had proposed *R. canescens* (Hedw.) Brid. instead. This change in type somewhat affected the nomenclature of infrageneric taxa within *Racomitrium* and subsequently influenced the naming of segregate genera derived from this heterogeneous and broadly circumscribed genus (Bednarek-Ochyra et al. 2014; Sawicki et al. 2015).

For the next quarter-century, bryologists largely overlooked the typification of generic names in Schimper’s (1860) opus. Zijlstra (2015) reignited the debate by claiming that the phrase on the book’s title page implies the figures represent typical species. This, however, is a clear overstatement: Schimper (1860) explicitly used the term **type** (*typus*), not **typical** (*typicus*). While it is possible that Schimper meant “exemplar”, not our modern nomenclatural type, that is irrelevant under the current wording of the "*Code*" (Turland et al. 2025). Notably, the same terminology appears in "*Nomenclator botanicus*" by Pfeiffer (1873a), who included a general statement on the unnumbered second page of the *Prefatio*: “*Species plantarum in libro meo omnino negliguntur, excepta indicatione illarum, quae****typum****generis novi aut novo modo circumscripti vel sectionis offerunt*” [= “Plant species are entirely neglected in my book, except for indication of those which are presented as the **type** of a new or re-circumscribed genus or of a section” (*emphasis ours*)]. Like Schimper (1860), Pfeiffer (1873a) clearly indicated type-species for generic or sectional names and these typifications are acceptable under the "*Code*" (Art. 7.11, Ex. 18; Turland et al. (2025)). Of course, it occasionally emerges that Schimper’s or Pfeiffer’s choices were erroneous or based on a misinterpretation of the protologue. However, such cases do not undermine the fundamental validity of their typifications. Similar issues occur even in contemporary practice and erroneous choices can be corrected in accordance with Art. 10 of the "*Code*" (Turland et al. 2025).

Typification of moss generic names by Schimper (1860) once again drew the attention of the Committee for Bryophytes in the early 2020s, during discussions surrounding the proposal to conserve the name *Oreas* Brid. (Brinda and Fedosov 2020). Although the name was formally proposed for conservation with the conserved type *Oreas martiana* (Hoppe & Hornsch.) Brid., based on the then-prevailing assumption that *Oreas* had first been typified by Pfeiffer (1873b) with “Weissia Mielichhoferi Schwägr.” (≡ *Weissia mielichhoferiana* Funck), the Committee ultimately accepted Schimper’s (1860) earlier typification of this generic name with *O. martiana* as effectively published (Klazenga 2023).

This decision was subsequently accepted by the General Committee with the suggestion that “bryologists should check whether this acceptance of Schimper’s typification in his 1860 paper has consequences for typifications of other names in that paper” (Wilson 2023). However, most of the issues arising from Schimper’s (1860) typifications, which had significantly affected the status of several of the aforementioned generic names, have in fact already been resolved via conservation proposals (Margadant and Geissler 1995).

Schimper’s (1860) typifications have recently gained broader recognition, as reflected in the TROPICOS database (https://www.tropicos.org/), where an attempt has been made to correctly list the types of all bryophyte genera. This includes the generic name *Neckera*, which was first typified with *N. pennata* (https://www.tropicos.org/name/35000848). In Table VI, Schimper (1860) presented an illustration labelled only with the name *Neckera*. However, the accompanying legend specifies *N. pennata* and includes captions identifying the illustrated details. Consequently, Dixon’s (1939) seemingly arbitrary selection of *N. pennata* aligns with Schimper’s earlier, but long-overlooked typification. This finding clarifies that Dixon’s (1939) choice was indeed correct and that Grout’s (1934) typification was not the earliest. Therefore, *N. pennata* should not be listed as a conserved type in Appendix III of the "*Code*", as suggested by Brinda and Davidse (2020), but rather its current status should be maintained. As noted above, *N. pennata* was treated as the conserved type of *Neckera* from the "*Montreal Code*" (Lanjouw et al. 1961) through to the "*Tokyo Code*" (Greuter et al. 1994). However, in the mid-1990s, W. D. Margadant re-discovered Schimper’s (1860) earlier typifications of many generic names, including *Neckera*. As a result, treating *N. pennata* as a conserved type became unnecessary. Accordingly, beginning with the "*Saint Louis Code*" (Greuter et al. 2000), the phrase "*typus conservandus*" associated with *N. pennata* was editorially removed.

### ﻿Synonyms of the generic name *Neckera*

In summary, the generic name *Neckera* currently has two heterotypic synonyms and ten homotypic synonyms (Brinda and Atwood 2025). The first heterotypic synonym is *Alsia* Sull., formerly treated as a monotypic genus endemic to the Pacific coast of North America (Schofield 1980). It included only *A. californica* (Hook. & Arn.) Sull., a species originally described as *Neckera californica* Hook. & Arn., which has recently been transferred back to *Neckera* (Olsson et al. 2011). The second is Neckera sect. Douglasiella (Kindb.) Paris, typified by the North American species *N. douglasii* Hook. Three of the homotypic synonyms are homonyms and seven represent names of homotypic infrageneric taxa. Of these, *Distichia* (Brid.) Brid. and *Braunia* Hornsch. are considered *nomina rejicienda*, while *Cryptopodium* Fürnr. is both a homonym and a superfluous name by typification. Additionally, *Cryptopodia* (Röhl.) Fürnr. is a superfluous name by typification and *Eleutera* P.Beauv. *ex* Stuntz is illegitimate, as it included the previously designated type of *Neckera*.

## ﻿Taxonomic and nomenclatural history of *Rhystophyllum*

### ﻿Earliest plant exsiccatae

**Figure 3.**
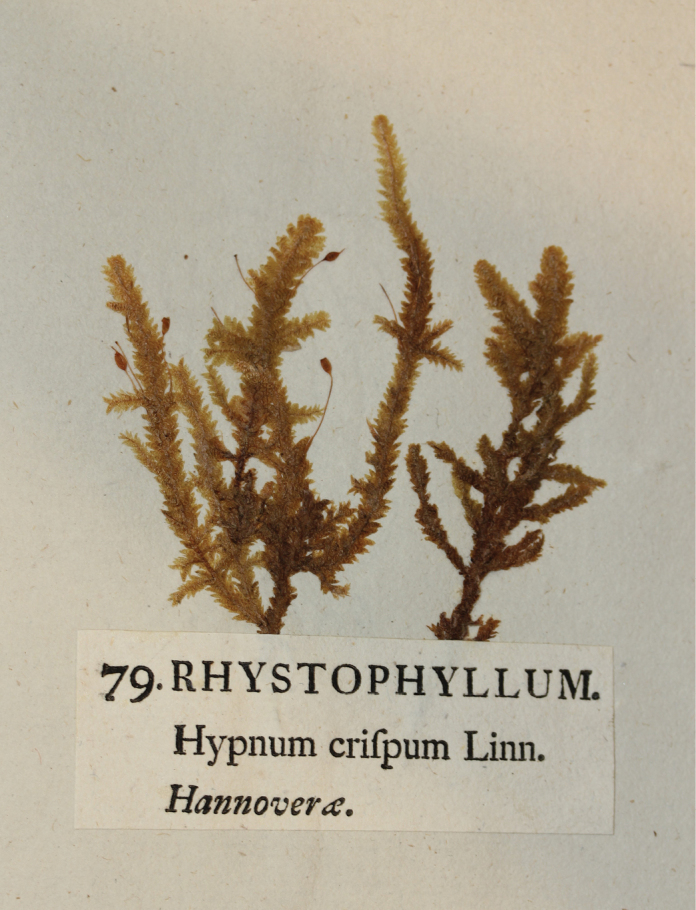
The specimen No. 79 from "*Phytophylacium Ehrhartianum*" from UPS with the unitary designation *Rhystophyllum* of *Neckera crispa* Hedw.

The name *Rhystophyllum* first appeared in bryological literature through the work of Jakob Friedrich Ehrhart (1780–1785, 1789), who used it on the label of a specimen distributed in 1780 as No. 79 in the eighth decade (a set of ten numbered specimens) of his exsiccata "*Phytophylacium Ehrhartianum*" (Fig. 3). Ehrhart (1742–1795), a German botanist of Swiss origin and a gifted student of Linnaeus, held the position of Royal Botanist in the Electorate of Hanover (Braunschweig-Lüneburg) during the final decade of his life. Ehrhart is best known in the history of botany as one of the pioneers in publishing exsiccatae, numbered collections of dried and pressed plant specimens, distributed under a common title, precisely identified and accompanied by printed labels, intended for circulation amongst botanists and institutions.

Although Johann Balthasar Ehrhart (1700–1756) is regarded as the first publisher of an exsiccata, his "*Herbarium vivum recens collectum*", issued in 1732, contained only medicinal plants and no cryptogams. Today, only three copies of this earliest exsiccata are known to exist (Stafleu 1972). On the other hand, Jakob Friedrich Ehrhart – the similarity of names is coincidental – published for commercial purposes seven series of exsiccatae in the years 1780–1793, covering about 1620 species of flowering and cryptogamic plants (Britten 1922, 1923; Stafleu and Cowan 1976; Sokoloff et al. 2002). For bryologists, the first two series are particularly important, the above-mentioned "*Phytophylacium Ehrhartianum*" and "*Plantae cryptogamae Linneae*" (Sayre 1969), comprising 320 specimens of mosses, liverworts, lichens, algae, club-mosses, horsetails and ferns, distributed in decades (Ehrhart 1785–1793, 1792). In the first of these series, only six species of mosses and ten lichens were distributed from the cryptogams. However, they are particularly interesting from a nomenclature perspective. On all labels, the standard Latin name of a species followed by a locality precede, as if in the form of a title, the specimen number and Latin uninomial on the top line (Fig. 3). Ehrhart (1789) calls them "nomina usualia", i.e. common names and treats them as an attempt to give each plant a distinct name that would be used independently of the binomial name. Although the author himself does not consider them equivalent to generic names, in later years, various authors began to consider them as such, although from a nomenclature perspective, they were typical "*nomina nuda*", as Ehrhart never published any diagnoses of them.

### ﻿Ehrhart’s unitary designations of species

The confusion surrounding generic names was addressed at the International Botanical Congress held in Montreal in 1959. The resulting "*Montreal Code*" (Lanjouw et al. 1961) expanded Article 20 by adding a note clarifying what should not be considered generic names. The second point of this note referred to “unitary designations of species”, exemplified by Ehrhart’s (1780–1785, 1789) names in "*Phytophylacium Ehrhartianum*", which imitated generic names. According to the "*Code*", such designations were to be rejected unless they have been validly published as generic names by subsequent authors. This provision remained in force until the Tokyo Congress in 1993, when a new Appendix V, "*Opera utique oppressa*", was introduced (Greuter et al. 1994). This Appendix listed publications considered suppressed works, meaning that names at specified ranks within them were not validly published. Amongst the works included was Ehrhart’s exsiccata "*Phytophylacium Ehrhartianum*" and its associated species index, published in 1789. As a result, Article 20.4, which had provided examples of names that should not be treated as generic names, was removed from the "*Code*". At the Melbourne Congress in 2012, Appendix V was renamed " Suppressed works" and re-designated as Appendix VI (Wiersema et al. 2015).

Of the six unitary designations for moss species listed in "*Phytophylacium Ehrhartianum*" (Ehrhart 1780–1785, 1789), only two, *Hippopodium* and *Tristichitis*, have fallen into complete obscurity. The former, applied to *Buxbaumia aphylla* Hedw. in the first decade under No. 10, was originally borrowed from Fabricius (1743). However, the generic name *Buxbaumia* Hedw. had already been introduced a year earlier by Haller (1742) and was subsequently accepted as the older name by Schmidel (1758) and Linnaeus (1760, 1763). Nevertheless, Röhling (1808) validated *Hippopodium* Röhl. as a generic name with *H. aphyllum* (Hedw.) Röhl. as its generitype. This makes *Hippopodium* homotypic with *Buxbaumia* which has nomenclatural priority. Despite evident priority of *Buxbaumia*, Röhling (1813) continued using *Hippopodium*, but since his death, this name has fallen into oblivion.

The designation *Tristichitis* was applied to *Mnium triquetrum* L. (≡ *Meesia triquetra* (L. *ex* Jolycl.) Ångstr.; see Ochyra (2001)), a species distributed in the sixth decade under No. 59. It was later only once considered by Röhling (1800) in a synopsis of the German moss genera, as a taxon close to *Meesia* Hedw. He provided a short diagnosis of *Tristichitis*, but no species were included. As a pre-starting point name, *Tristichitis* is not validly published and has been supplanted by the generic name *Meesia* Hedw. (Hedwig 1785–1787), which was validated in "*Species muscorum frondosorum*" (Hedwig 1801).

Finally, the unitary designation *Ampullaria* was assigned to *Splachnum ampullaceum* Hedw., published in the fifth decade under No. 49 (Ehrhart 1780–1785). Müller (1848) subsequently validly published this name as a section within *Splachnum* Hedw.; however, it is now superfluous given that Schimper (1860) selected *Splachnum ampullaceum* Hedw. as the type of this genus name.

Of the three remaining unitary designations, all have persisted to the present day, with two still in common use. The earliest legitimised name was *Diphyscium*, which Ehrhart (1780–1785) used to designate a specimen of *Webera diphyscium*, published in the tenth decade as No. 99. Notably, this binomial was first published in that work, not in Ehrhart’s (1779) earlier publication describing the genus *Webera*, as incorrectly reported by Limpricht (1894) and Wijk et al. (1969). Mohr (1803) later validated the generic name *Diphyscium*, assigning to it a single species: *D. foliosum* (Hedw.) D.Mohr. While Mohr acknowledged Ehrhart in his discussion: “*Itaque triviali Ehrharti vocem, tanquam substantivum, pro novo conficiendo generi nomine lubenter recepi*” [= “Therefore, I gladly accepted Ehrhart’s trivial word, as a substantive, for the newly-coined genus name”], he ascribed the generic name to himself alone as “*mihi*”.

The second unitary designation, *Paludella*, was established by Ehrhart (1780−1785) and remains in common use today. It was originally proposed for *Bryum squarrosum* L., a strikingly beautiful peatland moss, which was published as No. 69 in the seventh decade of his exsiccata. Initially, this species was placed in several broad genera, including *Bryum* Hedw., *Mnium* Hedw. and *Hypnum* Hedw. Eventually, Bridel (1817) transferred it to the monotypic genus *Paludella* Ehrh. *ex* Brid., adopting the uninomial Ehrhart had introduced 37 years earlier.

### ﻿Validation of *Rhystophyllum*

The third and final unitary designation that has withstood the test of time is *Rhystophyllum*. Ehrhart (1780–1785) originally proposed this name for the calciphilous moss *Hypnum crispum*, which is widely distributed throughout Europe (Ochyra et al. 1988). He included it as No. 79 in the eighth decade of his first exsiccata, "*Phytophylacium Ehrhartianum*" (Fig. 3). The name was later revived by Müller (1850), who adopted it for one of two subsections within *Neckera* sect. ‘Euneckera ’. In defining subsect. Rhystophyllum Müll. Hal., Müller provided a brief diagnosis − "*Folia transversim rugosa*" − to distinguish it from subsect. Leiophyllum Müll.Hal., whose species possess "*Folia laevia, haud transversim rugulosa*". Subsect. Rhystophyllum was subsequently elevated to the rank of section, though there is considerable confusion regarding the identity of the author, as well as the date and place of publication. According to the *Index muscorum* (Wijk et al. 1964) and Crosby (1968), the name was validated by Braithwaite (1905). However, the elevation of subsect. Rhystophyllum to the sectional rank was actually carried out much earlier by Mitten (1869).

The "*Index muscorum*" (Wijk et al. 1964) suggests that Lindberg (1879) most likely treated this taxon as a subgenus of *Neckera*. According to the compilers of this compendium, although this author does not explicitly indicate the rank of the subdivision in that publication, they assume that all subdivisions of genera designated by capital letters are most probably subgenera. Indeed, Lindberg (1879) does not define the rank of these subdivisions in the main text. It is only in the final chapter, entitled "*Comparatio denominationum in editione decima Florae Hartmanianae cum iisdem in hoc opere nostro datis*" (pp. 45–48), that he clearly refers to them as sections. For example, *Racomitrium* [in Hartman (1871)] corresponds to *Grimmia***sect. A** [in Lindberg’s work] (*emphasis ours*). Thus, following Mitten (1869) and Bescherelle (1872), this constitutes the third isonym of Neckera sect. Rhystophyllum (Müll.Hal.) Mitt. Formally, the status of *Rhystophyllum* as a subgenus of *Neckera* should be attributed to the compilers of the "*Index muscorum*", as reflected in the TROPICOS database [https://www.tropicos.org/name/35171285], where it is cited as Neckera subg. Rhystophyllum (Müll.Hal.) Lindb. *ex* Wijk, Margad. & Florsch. However, this is an invalid name, erroneously used for Neckera sect. Rhystophyllum.

Given that various genera proposed by Ehrhart were previously accepted and that Linnaeus’s (1753) "*Species plantarum*" was still the starting point for moss nomenclature at the time, Britton (1905) concluded that *Rhystophyllum* also merited recognition as a distinct genus and that it predated *Neckera*. Although she did not acknowledge that Ehrhart (1780–1785) failed to provide a formal description or diagnosis of this “genus”, she nonetheless included a very brief diagnosis on page 4 of her text: “…as Ehrhart’s genus *Rhystophyllum* is monotypic, […], there is no question as to its meaning or the application of the name, seeing that its derivation from two Greek words meaning ‘**Wrinkled-Leaved’ indicates one of the most noticeable characters of the genus** as limited in modern times” (*emphasis ours*). This single morphological trait may be accepted as sufficient to serve as a validating description of the genus. In fact, this character was also used by Müller (1850) when describing subsect. Rhystophyllum. As the requirements for valid publication of *Rhystophyllum* are fulfilled in Britton’s (1905) paper, it may be considered a cryptic reference to the subsectional name Neckera subsect. Rhystophyllum as used by Müller (1850: p. 46), in accordance with Art. 41.4 of the "*Code*" (Turland et al. 2025). Notably, the citation accompanying the genus name *Rhystophyllum* − “Crypt. Exsic. No. 97. 1780” − constitutes an indirect reference to Müller’s (1850) work, as it even reproduces his error in the cited specimen number: “No. 97” should correctly read “No. 79”.

Britton (1905) considered *Hypnum crispum* (≡ *Neckera crispa*) to be the generitype of *Rhystophyllum* as well as Hedwig’s *Neckera*. She was, therefore, in agreement with Grout’s erstwhile selection for *Neckera*, but in conflict with Schimper’s. She was likely unaware that Pfeiffer (1874) had previously typified Müller’s subsect. Rhystophyllum with the same species, but this merely reinforces the interpretation that Britton’s *Rhystophyllum* should be treated as a status nova rather than the name of a new taxon or a nomen novum for *Neckera*. Given that Britton’s *Rhystophyllum* has a legitimate basionym with a type that is different from the type of *Neckera*, the name must be considered legitimate even though Britton incorrectly included the previously designated type of *Neckera* in her circumscription (Art. 52.4, Turland et al. (2025)). For most of the last century, *Neckera* has been treated as a large, heterogeneous genus typified by *N. pennata* and *Rhystophyllum* was considered a heterotypic synonym (Farr et al. 1979b). However, following the division of *Neckera* into three segregate genera, *Neckera* s.str., *Exsertotheca* and *Alleniella* (Olsson et al. 2011), *Rhystophyllum* (Müll.Hal.) Ehrh. *ex* E.Britton should be recognised as the correct name for *Exsertotheca*. Both names are homotypic synonyms typified by *Neckera crispa*, but *Rhystophyllum* has nomenclatural priority.

The second segregate of *Neckera*, *Alleniella*, also has a related subsectional name. In addition to subsection Rhystophyllum, which includes species with distinctly undulate leaves, Müller (1850) recognised subsection Leiophyllum, comprising species with smooth, non-undulate leaves. According to Art. 10.8 of the "*Code*" (Turland et al. 2025), this subsection is automatically typified by *Neckera leiophylla* W.Gümbel *ex* Müll.Hal. This species is conspecific with *N. besseri* (Łobarz.) Trevis., which is the type species of *Alleniella*. However, elevating subsect. Leiophyllum to the rank of genus is pre-empted by *Leiophyllum* (Pers.) R.Hedw. (1806, Ericaceae), so it may only be used at the infrageneric ranks.

## ﻿Nomenclatural implications

### ﻿ Neckera

Taxon classificationPlantaeHypnalesNeckeraceae

Hedw., Spec. Musc. Frond.: 200. 1801 nom. cons.

0EE0A593-8384-5741-A379-7309C513D74C

≡ Neckera [unranked] Cryptopodia Röhl., Borkhausen’s Ringen: 148. 1808 ≡ Neckera [unranked] Distichia Brid., Muscol. Recent. Suppl. 4: xvi, 137. 1819 ≡ Distichia Brid., Bryol. Univ. 1: xxxvii. 1826; 2: 238, 757, 787, 811. 1827 ≡ Neckera subg. Distichia (Brid.) Brid., Bryol. Univ. 2: 238, 757. 1827 ≡ Cryptopodium Fürnr., Flora 12(2, Beil. 1): 81. 1827 ≡ Braunia Hornsch., Jahrb. Wiss. Krit. 1828(59/60): 467. 1828, *nom. illeg*. ≡ Neckera subg. Cryptopodia (Röhl.) Rchb., Consp., Regn. Veg. 1: 33. 1828 ≡ Cryptopodia (Röhl.) Fürnr., Flora 12(2, Ergänzugsbl.): 49. 1829 ≡ Neckera sect. Distichia (Brid.) Müll.Hal., Linnaea 18: 707. 1844 ≡ Eleutera P.Beauv. *ex* Stuntz, Bull. Torrey Bot. Club 27: 202. 1900, *nom. illeg*. ≡ Neckera sect. Cryptopodia (Röhl.) Broth. in Engl. & Prantl, Nat. Pflanzenfam. 1(3): 843. 1906. Typus: Neckera pennata (*vide*Schimper 1860: t.p., 732).
= Alsia Sull., Not. Sp. Moss.: 16. 1855. Typus: Alsia californica (Hook. & Arn.) Sull. (Neckera californica Hook. & Arn.)
= Neckera [unranked] Douglasiella Kindb., Eur. N. Amer. Bryin. 1: 15. 1897 ≡ Neckera sect. Douglasiella (Kindb.) Paris, Index Bryol. Suppl.: 132. 1900. Typus: Neckera douglasii Hook. (*vide*Wijk et al. 1964: 432).

### ﻿ Rhystophyllum

Taxon classificationPlantaeHypnalesNeckeraceae

(Müll.Hal.) Ehrh. ex E.Britton, Bryologist 8(1): 4−5. 27 Dec 1904

0ABC4A82-5736-5CC6-A22B-D105D9F382FA

≡ Neckera subsect. Rhystophyllum Müll.Hal., Syn. Musc. Frond. 2: 46. Sept 1850 ≡ Neckera sect. Rhystophyllum (Müll.Hal.) Mitt., J. Linn. Soc. Bot. 12: 453. 1869 ≡ Neckera subg. Rhystophyllum (Müll.Hal.) Wijk, Margad. & Florsch., Regnum Veg. 33: 432. 1964, *nom. inval. err. pro sect*. ≡ Exsertotheca S.Olsson, Enroth & D.Quandt, Taxon 60(1): 45. 2011. Typus: Rhystophyllum crispum (Hedw.) Ochyra, Plášek & Brinda (Neckera crispa Hedw., *vide*Pfeiffer 1874: 965).

### ﻿ Rhystophyllum baeticum

Taxon classificationPlantaeHypnalesNeckeraceae

(J.Guerra, J.F.Jiménez & J.A.Jiménez) Ochyra, Plášek & Brinda comb. nov.

6EBB17B2-F6D0-5B93-B932-F273986ADEA3

#### Basionym.

*Neckera baetica* J.Guerra, J.F.Jiménez & J.A.Jiménez, Nova Hedwigia 91: 259. 2010.

### ﻿ Rhystophyllum crispum

Taxon classificationPlantaeHypnalesNeckeraceae

(Hedw.) Ochyra, Plášek & Brinda comb. nov.

232BC628-A1CB-55F9-995F-919F83CCD13D

#### Basionym.

*Neckera crispa* Hedw., Spec. Musc. Frond.: 206. 1801.

### ﻿ Rhystophyllum intermedium

Taxon classificationPlantaeHypnalesNeckeraceae

(Brid.) Ochyra, Plášek & Brinda comb. nov.

65FF4B75-3A02-5113-9EB0-88E7A0A7DF62

#### Basionym.

*Neckera intermedia* Brid., Muscol. Recent. Suppl. 2: 24. 1812.

### ﻿ Alleniella

Taxon classificationPlantaeHypnalesNeckeraceae

S.Olsson, Enroth & D.Quandt, Taxon 60(1): 45. 2011

684DF9ED-73A7-5191-846A-AC4C1B29C1BF

= Leskea [unranked] Complanatae Brid., Muscol. Recent. Suppl. 2: 50. 1812 ≡ Hypnum sect. Complanata (Brid.) Arn., Disp. Méth. Mousses: 58. 1825. Typus: Leskea complanata Hedw., syn. nov.
= Neckera subsect. Leiophyllum Müll.Hal., Syn. Musc. Frond. 2: 41. Sept 1850 ≡ Neckera sect. Leiophyllum (Müll.Hal.) Lindb., Musci Scand.: 40. 1879 ≡ Neckera subg. Leiophyllum (Müll.Hal.) Lindb. *ex* Wijk, Margad. & Florsch., Regnum Veg. 33: 432. 1964, *nom. inval. err. pro sect*. Typus: Neckera leiophylla W.Gümbel. *ex* Müll.Hal. [= Alleniella besseri (Łobarz.) S.Olsson, Enroth & D.Quandt], syn. nov.

## Supplementary Material

XML Treatment for Neckera

XML Treatment for Rhystophyllum

XML Treatment for Rhystophyllum baeticum

XML Treatment for Rhystophyllum crispum

XML Treatment for Rhystophyllum intermedium

XML Treatment for Alleniella

## ﻿Additional information

### Use of AI

No use of AI was reported.

### Author ORCIDs

Ryszard Ochyra https://orcid.org/0000-0002-2541-0722

Vítězslav Plášek https://orcid.org/0000-0002-4664-2135

John C. Brinda https://orcid.org/0000-0001-9083-1235

### Data availability

All of the data that support the findings of this study are available in the main text.
